# Evaluation of the Autologous Genetically Enriched Leucoconcentrate on the Lumbar Spinal Cord Morpho-Functional Recovery in a Mini Pig with Thoracic Spine Contusion Injury

**DOI:** 10.3390/biomedicines11051331

**Published:** 2023-04-30

**Authors:** Ravil Garifulin, Maria Davleeva, Andrei Izmailov, Filip Fadeev, Vage Markosyan, Roman Shevchenko, Irina Minyazeva, Tagir Minekayev, Igor Lavrov, Rustem Islamov

**Affiliations:** 1 Department of Histology, Cytology and Embryology, Kazan State Medical University, 420012 Kazan, Russia; 2 Department of Neurology, Mayo Clinic, Rochester, MN 55905, USA

**Keywords:** spinal cord injuries, swine, autologous blood transfusion, leukocytes, genetic vectors, genetic therapy, vascular endothelial growth factor A, glial cell line-derived neurotrophic factor, neural cell adhesion molecules, spinal cord regeneration

## Abstract

Background: Pathological changes associated with spinal cord injury (SCI) can be observed distant, rostral, or caudal to the epicenter of injury. These remote areas represent important therapeutic targets for post-traumatic spinal cord repair. The present study aimed to investigate the following in relation to SCI: distant changes in the spinal cord, peripheral nerve, and muscles. Methods: The changes in the spinal cord, the tibial nerve, and the hind limb muscles were evaluated in control SCI animals and after intravenous infusion of autologous leucoconcentrate enriched with genes encoding neuroprotective factors (VEGF, GDNF, and NCAM), which previously demonstrated a positive effect on post-traumatic restoration. Results: Two months after thoracic contusion in the treated mini pigs, a positive remodeling of the macro- and microglial cells, expression of PSD95 and Chat in the lumbar spinal cord, and preservation of the number and morphological characteristics of the myelinated fibers in the tibial nerve were observed and were aligned with hind limb motor recovery and reduced soleus muscle atrophy. Conclusion: Here, we show the positive effect of autologous genetically enriched leucoconcentrate-producing recombinant neuroprotective factors on targets distant to the primary lesion site in mini pigs with SCI. These findings open new perspectives for the therapy of SCI.

## 1. Introduction

The functional restoration and effect of rehabilitation after thoracic SCI depends on the morpho-functional state of the lumbar spinal cord, distant from the epicenter of the injury. Disruptions of the descending and ascending pathways interrupt the neural connections between the brain and spinal cord neurons innervating target organs, leading to motor and sensory dysfunctions [1,2]. Lumbar α-motoneurons deprived of efferent signaling from the pyramidal and extrapyramidal systems lose control of the skeletal muscles of the hind limbs, which depend on the impulse activity of motor neurons and neurogenic molecules released from the nerve terminal at the neuromuscular junction [3]. In addition to post-traumatic changes, SCI causes neuroinflammation-related damage to segments of the spinal cord remote from the epicenter of injury. Numerous alterations in the lumbar region were found after thoracic SCI, with changes in the number of neurons [4,5,6], synapses [6,7], as well as in the response of microglia [8,9,10,11] and astrocytes [12,13]. Thus, pathological changes in SCI affect large areas around the epicenter of the lesion [8,14,15]; therefore, after thoracic SCI, the distant areas, i.e., lumbosacral segments of the spinal cord, are important therapeutic targets.

Currently, the most promising therapeutic regenerative strategies for SCI rely on cell and gene therapies, small biomolecules, biodegradable materials, and neuromodulation [16]. Numerous novel regenerative approaches are under intensive preclinical research. At the same time, only a few therapeutic strategies were advanced to clinical trials and focused on the following areas: (i) neurotransplantation of mesenchymal stem cells (MSC) derived from the umbilical cord, adipose tissue, bone marrow, olfactory ensheathing cells (OEC), and Schwann cells [17,18,19], (ii) administration of small biomolecules neutralizing inhibitors of neuroregeneration, such as the Rho-associated protein kinase (ROCK) inhibitor and anti-NOGO (neurite outgrowth inhibitor) monoclonal antibody [20,21], and (iii) implantation of neuro-spinal scaffolds [22,23]. Unfortunately, these clinical studies showed minimal or no progress and are still far from practical implementation [21,22]. The other innovative cell-based approach in preclinical studies of SCI treatment is based on the application of exosomes derived from MSC and neural stem cells [24]. Preclinical studies demonstrated promising results in using engineered exosomes carrying microRNA derived from gene-modified MSC [25]. However, future clinical trials will require compliance with good manufacturing practice to ensure a standardized protocol of exosome biogenesis, purification and quality control [26].

Delivery of therapeutic genes expressing growth, neurotrophic, anti-apoptotic, and anti-inflammatory factors to the injured spinal cord using viral vectors (in vivo gene therapy) or cell carriers (ex vivo gene therapy) has demonstrated positive effects on post-traumatic spinal cord regeneration in various animal models [27,28]. However, to date, no gene therapy in vivo or ex vivo has been tested in clinical trials. A long list of potential genes applicable for stimulation of post-traumatic neuroregeneration, and the variety of viral vectors used to target cell transduction and delivered genetic constructs create enormous variations, which slow down the progress in the field of gene therapies [29,30]. Moreover, testing these therapies on large animals should be considered before translation to humans, as the differences in regeneration capacity and anatomy between small and large animals impact the therapeutic outcome [31,32]. A recently developed approach with autologous genetically enriched leucoconcentrate simultaneously producing recombinant vascular endothelial growth factor (VEGF), glial cell line-derived neurotrophic factor (GDNF), and neural cell adhesion molecule (NCAM) demonstrated functional and morphological improvement in SCI mini pigs [33] similarly to previously reported results using other cell-mediated therapies [34]. The positive effect of the genetically enriched leucoconcentrate on the molecular and cellular changes was found in the epicenter and adjacent supra- and sublesion parts of the spinal cord after contusion injury at Th8-Th9 [33]. Intravenous infusion of the autologous leucoconcentrate producing recombinant VEGF, GDNF, and NCAM in the acute phase of spinal cord injury demonstrated increased survivability of the spinal cord cells, recovery of synaptic protein expression, prevention of astrogliosis in the grey matter and higher growth rates of regenerating axons accompanied by a higher number of oligodendroglial cells in the lateral corticospinal tract region in the white matter. Following these reports, we expanded our study on thoracic SCI mini pigs treated with autologous leucoconcentrate enriched with genes encoding VEGF, GDNF, and NCAM [33] to evaluate the recovery of the lumbar spinal cord.

**Figure 1:**
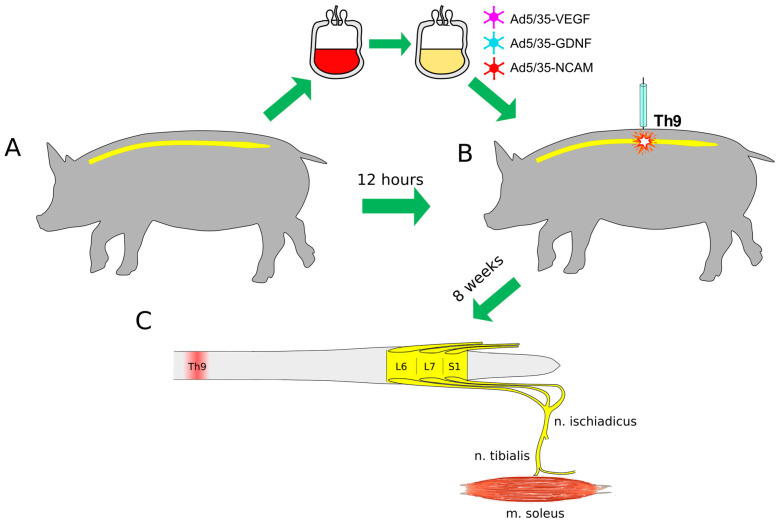
Study design. (**A**)—Collection of 50 mL of venous blood and preparation of the autologous genetically enriched leucoconcentrate. (**B**)—Spinal cord contusion injury at the Th9 segment followed by intravenous autoinfusion of the genetically enriched leucoconcentrate 4 h after surgery. At 2, 4, and 8 weeks after surgery, behavioral tests and electrophysiological studies were performed. (**C**)—The lumbar spinal cords (L6-S1), tibial nerves and soleus muscles were harvested at 8 weeks after injury. Ad5/35—adenovirus serotype 5 with fibers derived from adenovirus serotype 35 fiber gene; VEGF—vascular endothelial growth factor; GDNF—glial cell line-derived neurotrophic factor; NCAM—neural cell adhesion molecule.

In this study, molecular and cellular changes in the lumbar spinal cord and peripheral nerves were evaluated in relation to restoration of motor performance and electrophysiological and histological examination of the hind limb skeletal muscles in mini pigs with thoracic SCI after intravenous infusion of the autologous genetically enriched leucoconcentrate simultaneously producing recombinant VEGF, GDNF, and NCAM (Figure 1). 

## 2. Materials and Methods

This study aligns with our long-term efforts to stimulate post-traumatic regeneration after spinal cord injury (SCI) [35]. To test our hypothesis that autologous leucoconcentrate enriched with *vegf165, gdnf, ncam1* may have positive effects on therapeutic targets distant to the primary lesion site after SCI, assuming its paracrine mechanism of action after intravenous infusion [36], in support of this concept, after low thoracic SCI, we evaluated the efficacy of the autologous genetically enriched leucoconcentrate on the lumbar spine, peripheral nerves, and hind limb skeletal muscles’ morpho-functional characteristics in relation with our previous studies [33,37]. 

Female adult miniature Vietnamese pot-bellied pigs (25–30 kg; *n* = 11) were used in the study. Two weeks before investigation, mini pigs were kept one per housing area in a 12 h light/dark regimen at a temperature of 24–25 °C with controlled air conditioning and properly organized access to food and water. Animal treatments were conducted according to the Animal Care and Use Committee of Kazan State Medical University (approval No. 5, dated 26 May 2020). Preparation of the autologous genetically enriched leucoconcentrate (GEL) and treatment of the animals are presented briefly, in view of the detailed descriptions of those in our recent publication, in which the same animals were employed [33]. 

### 2.1. Preparation of the Autologous Genetically Enriched Leucoconcentrate

Recombinant replication-defective viral vectors carrying *vegf165, gdnf,* and *ncam1* were constructed based on the human adenovirus serotype 5 with fibers derived from adenovirus serotype 35 fiber gene (Ad5/35) and nucleotide sequences encoding VEGF165 (Gene Bank NM_001171626.1), GDNF (Gene Bank NM_000514.4), and NCAM1 (Gene Bank NM_001076682.2) [37]. In the study, we used Ad5/35 to provide effective transduction of leucocytes in the blood bag via the 35 fiber, which has a high affinity for the cluster of differentiation 46 (CD46) that is expressed on all leucocytes [36]. Transduction of the leucoconcentrate directly in the blood bag is a safe and economic approach that prevents usage of antibiotics and biological materials of animal origin. 

From each experimental mini pig, 50 mL of peripheral blood was collected in a plastic blood bag from v. subclavia in a 14–16 h period before SCI modelling. The procedure for leucoconcentrate preparation was performed according to our original protocol [36] and subsequently included sedimentation of erythrocytes with 6% hydroxyethyl starch, centrifugation at 34× *g* for 10 min at 10 °C (DP-2065 R PLUS, Centrifugal Presvac RV; Presvac, Buenos Aires, Argentina) and washing with saline. The obtained leucoconcentrate was immediately transduced with a mixture of chimeric adenoviral vectors carrying cDNA encoding VEGF165, GDNF, and NCAM1. Transduction was performed according to the nucleated cell count in the leucoconcentrate, the titers and the equal ratio of each viral vector correspondingly: 1/3 Ad5/35-VEGF165 (2.0 × 10^9^ PFU/mL), 1/3 Ad5/35-GDNF (7.0 × 10^10^ PFU/mL), and 1/3 Ad5/35-NCAM1 (5.0 × 10^10^ PFU/mL) for 12 h in the blood bag with a multiplicity of infection (MOI) equal to 10 [37]. After washing with saline and centrifugation, the solution (30 mL) in the blood bag was considered as the leucoconcentrate enriched with *vegf165, gdnf* and *ncam1* (GEL-VGN). The efficacy of the leucocyte transduction was confirmed as described earlier [33,37]. In vitro comparative molecular analysis of naïve and gene-modified leucocytes 72 h after culturing confirmed effective expression of *vegf165, gdnf* and *ncam1* by RT-PCR (synthesis of transgenes mRNA), immunofluorescence method (synthesis of the recombinant VEGF, GDNF, and NCAM), and ELISE (secretion of recombinant VEGF, GDNG, and NCAM by leucocytes in the culture medium).

### 2.2. Animals Treatment

A moderate contusion injury of the spinal cord was performed a day after blood collection for GEL preparation. SCI was carried out under deep anesthesia induced by intramuscular administration of Zoletil 100 (Virbac Sante Animale, Carros, France) at a dose of 10 mg/kg and maintained using an inhalation apparatus (Minor Vet Optima, Zoomed, Moscow, Russia) with isoflurane (Laboratorios Karizoo, S.A., Barcelona, Spain) as a 2.0–2.5% mixture with oxygen. After laminectomy at the Th8-Th9 vertebral level, the dura matter was exposed, and a 50 g metal rod with a diameter of 9 mm was dropped on the spinal cord from a 50 cm height as described in our previous study [34]. Under anesthesia, the mini pigs were kept on the operating table with a plate maintaining their body temperature at 38 °C, and 4 h after SCI, the mini pigs in the therapeutic group (*n* = 4), were infused via the auricular vein with 30 mL of the genetically enriched autologous leucoconcentrate. Animals from the control group (*n* = 4) received an infusion of the naïve autologous leucoconcentrate (Figure 1). Intact (healthy) mini pigs (*n* = 3) were employed to collect basic electrophysiological and histological data for comparative analysis of the morpho-functional changes in experimental animals (Figure 1). The rationale for using only autologous leucoconcentrate in control experiments was to exclude the influence on neuroregeneration by naïve leukocytes used as cell carriers for the delivery of the therapeutic genes. Based on our previous comparative analysis, which showed no impact of the direct or leucocyte-mediated delivery of the adenoviral vector carrying reporter gfp on the spinal cord recovery [38], in this study we did not employ these control groups.

### 2.3. Behavioral Assessment

Post-traumatic recovery of motor functions in mini pigs with SCI was evaluated using the Porcine Thoracic Injury Behaviour Scale (PTIBS) [39]. Behavioral assessment was performed when the experimental animal was placed in the center of the arena (2.5 m × 2.5 m) and evaluated according to the 10-point PTIBS score. In general, scores of 1 to 3 indicated "hind limb dragging", the scores of 4 to 6 corresponded to various degrees of "stepping", and the scores of 7–10 were related to "walking" ability. The PTIBS score was estimated by two observers in a blinded manner with respect to the experimental groups a week before SCI modeling and 2, 4, and 8 weeks after surgery.

### 2.4. Electrophysiological Study

Before the electrophysiological assessment, mini pigs were anesthetized and connected to an inhalation apparatus, as described above. A Digitimer DS7A (Digitimer Ltd., Welwyn Garden, UK), amplifier with filters ranging from 5 Hz to 2 kHz (Biosignal amplifier, g.tec medical engineering GmbH, Schieldberg, Austria) and LabChart data collection and analysis systems (AD Instruments Inc., Colorado Springs, CO, USA) were used to evaluate M-response in the soleus muscle of both hind limbs evoked by electrical stimulation of the sciatic nerve. Stimulating stainless steel electrodes (0.6 mm in diameter and 50 mm length) were inserted into the area of the sciatic nerve projection at 2 cm below the large trochanter of the femur. M-response was recorded using the similar needle electrodes injected into the soleus muscle before and 2, 4, and 8 weeks after SCI. Stimulation was performed with a single rectangular pulse with a frequency of 0.6 Hz, duration of 0.2 ms, and a current range of 4–72 mA. The parameters of M-responses were obtained by stimulating the sciatic nerve with various current intensities. The absolute values of the duration and amplitude of the M-responses were averaged in each animal by current intensity and then averaged per group. Data are presented as a percentage for comparison with intact animals, the absolute values of which were taken as 100%.

### 2.5. Sample Collection

Animals from the therapeutic and control groups were sacrificed 60 days after SCI. The spinal cord was removed from the vertebral column, and the lumbar part was fixed in 4% paraformaldehyde (Sigma, St. Louis, MO, USA) in phosphate-buffered saline (pH 7.4) and processed for immunofluorescence staining. The 5 mm long fragments from both tibial nerves were taken, fixed in 2.5% solution of glutaraldehyde for 4 h, incubated in 1% solution of osmium tetroxide for 24 h, and embedded in EMbed 812 (Electron Microscopy Sciences, Hatfield, PA, USA) for preparation of the semi-thin sections. Skeletal muscles (*m. soleus*) from both hind limbs were weighed, fixed in 4% paraformaldehyde and processed for immunofluorescence phenotyping and morphometric analysis of the skeletal muscle fibers.

### 2.6. Immunofluorescence Study of Lumbar Spinal Cord

Frozen free-floating cross-sections of 20 μm thickness were prepared from L6-S1 level with a cryostat (Microm HM 560, Thermo Scientific, Waltham, MA, USA). Antibodies (Ab) against potassium-chloride cotransporter protein (KCC2), choline acetyltransferase (Chat), synaptophysin and postsynaptic density protein of 95 kDa (PSD95) were employed to analyze functional activity of the motor neurons. Astrocytes and oligodendroglial and microglial cells were identified with Abs to glial fibrillary acidic protein (GFAP), oligodendrocyte transcription factor (Olig2), and ionized calcium binding adaptor molecule 1 (Iba1), correspondingly. The appropriate secondary Abs were used to visualize the target molecules (Table 1). Nuclei were counterstained with DAPI (10 μg/mL in PBS, Sigma, Burlington, MA, USA), and sections were embedded in glycerol (GalenoPharm, Saint Petersburg, Russia). The slides were investigated with a luminescence microscope Axioscope A1 (Carl Zeiss, Oberkochen, Germany) and a confocal microscope Leica TCS SP5 MP (Leica Microsystems, Wetzlar, Germany) using identical settings. Obtained digital images were analyzed using ImageJ (NIH) software. Expression of the target molecules was evaluated in the ventral and dorsal horns with an area of 0.05 mm^2^. The count of Olig2-positive cells was performed in regard to specific nuclear immunostaining and DAPI nuclear counterstaining. Expression of neural (KCC2, Chat, Synaptophysin, PSD95) and glial markers (GFAP, Iba1) was estimated as the relative immunopositive area and presented as percentage considering the studied area [37].

### 2.7. Morphometric Analysis of the Sciatic Nerve Myelinated Fibers

Semi-thin cross-sections of the tibial nerves from both hind limbs were cut using an ultramicrotome (LKB-3; LKB, Sollentuna, Sweden) and stained with methylene blue dye. Digitized images of the tibial nerves were collected for morphometric analysis. Total count of myelinated fibers, thickness of the myelin sheet and axon diameter were measured in an area of 0.05 mm^2^ in each sample using ImageJ (NIH) software.

### 2.8. Hind Limb Skeletal Muscle Study

Slow soleus muscles were investigated from both hind limbs of the experimental animals. Phenotyping of the skeletal muscle fibers was performed using immunofluorescence staining of the 10 μm frozen cross-sections with Ab to slow myosin heavy chains. The number of slow skeletal muscles was expressed as percentage in regard to the total count of 200 muscle fibers in each studied muscle. Morphometric analysis of the skeletal muscle fibers area was performed in digital images captured with a 20-fold magnification of the microscope. The area of 200 skeletal muscle fibers in each soleus muscle was measured using the ImageJ (NIH) software.

### 2.9. Statistics

Statistical data analysis and visualizations were performed using R version 4.1.2 (R Foundation for Statistical Computing, Vienna, Austria). Sample distributions of quantitative values were visualized using box plots, and descriptive statistics are presented as: (median [1st quartile; 3rd quartile]). The results of M-response were presented as mean ± SEM. The Kruskal–Wallis test was used to compare experimental groups. Dunn’s test was used as the post hoc method. Differences were considered statistically significant, where *p* < 0.05.

## 3. Results

### 3.1. Hind Limb Skeletal Muscle Recovery 

Behavioral tests and electrophysiological and histological studies were performed to evaluate the recovery of the hind limb skeletal muscles innervated by lumbar motor neurons.

#### 3.1.1. Motor Activity

**Figure 2:**
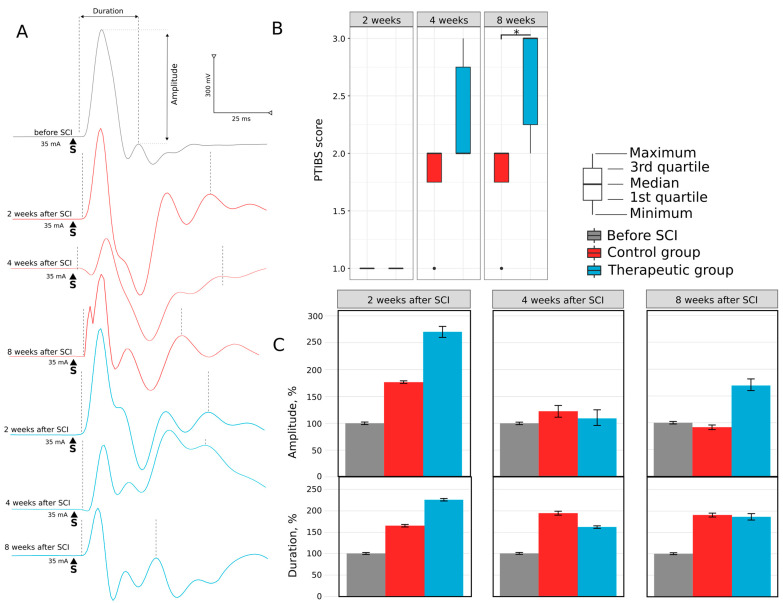
Hind limb skeletal muscles recovery in mini pigs after low thoracic spinal cord injury (SCI). (**A**)—Motor activity of mini pigs in control and therapeutic groups at 2, 4, and 8 weeks after SCI assessed with the Porcine Thoracic Injury Behavioural Scale (PTIBS). (**B**)—Evaluation of amplitude and duration of motor-evoked potential (M-response) in the soleus muscle evoked by electrical stimulation of the sciatic nerve in control and treated mini pigs at 2, 4, and 8 weeks after SCI, *—*p* < 0.05. (**C**)—Analysis of the amplitude and duration of motor-evoked potential.

Two weeks after SCI, control and treated mini pigs demonstrated a drastic decline in motor activity. PTIBS score was equal to 1 point, animals were not able to stand up, and the sacrum and knees of the hind limbs were on the floor (Figure 2A). Four weeks after SCI, the PTIBS score increased to 2.0 (2.0; 2.75) points in the therapeutic group; the animals tried to pull their hind limbs to the belly and stand on them. The control mini pigs were trying to move their hind limbs by this time; the PTIBS score was 2.0 (1.75; 2.0). At 8 weeks, a further increase in motor activity was observed in the therapeutic group, with the PTIBS score corresponding to 3.0 (2.25; 3.0). Treated animals demonstrated active flexion in hind limbs’ hips, knees, and ankles, and mini pigs could stand on four legs and tried to make steps. Control animals at 8 weeks post-SCI could drag their hind limbs while moving with their front limbs; the PTIBS score (2.0 (1.75; 2.0)) was lower than in the therapeutic group (*p* = 0.009). The recovery of locomotor activity was consistent with the results of our pilot study in SCI pigs treated with genetically modified human umbilical cord blood cells overexpressing VEGF, GDNF, and NCAM [40].

#### 3.1.2. Electrophysiology

The soleus muscle response to electrical stimulation of the sciatic nerve at 2, 4, and 8 weeks after SCI revealed the changes in the pattern of M-response in experimental animals (Figure 2B). In control mini pigs, M-response had a polyphasic shape and was associated with an increased duration at 2 (165 ± 7.1%), 4 (194 ± 11%) and 8 (190 ± 8.5%) weeks after SCI (Figure 2C). In the treated animals, the M-response also had a multiphase pattern with increased duration at 2 (225 ± 6%), 4 (161 ± 5.9%), and 8 (185 ± 15.7%) weeks after SCI. The amplitude of M-response was increased 2 weeks after SCI both in control (175 ± 4.6%) and treated (274 ± 19.4%) animals. At the following 4 and 8 weeks, the amplitude in the control group decreased to 121 ± 13.6% and 91 ± 6.3%, and in the therapeutic group to 108 ± 22.6% and 169 ± 15%, correspondingly. The threshold and latency in all intact and experimental animals were not significantly different.

#### 3.1.3. Histology

**Figure 3:**
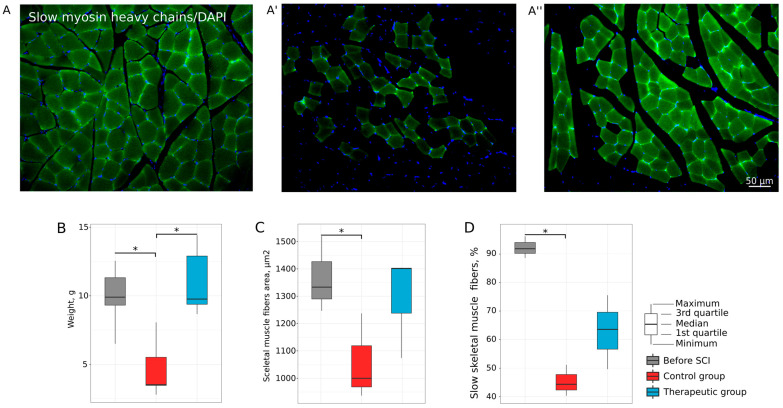
Assessment of soleus muscles in mini pigs 8 weeks after low thoracic spinal cord injury (SCI). (**A**,**A′**,**A″**) Cross-sections of the muscle stained with antibodies against slow myosin heavy chains (green glow) from intact, control, and therapeutic groups, correspondingly. Nuclei were counterstained with DAPI (blue glow). (**B**) Soleus muscle weighing analysis. (**C**) Morphometric analysis of skeletal muscle fiber area. (**D**) Relative content of slow skeletal muscle fibers, *—*p* < 0.05.

At 8 weeks after SCI, the raw weight (g) of soleus muscles in the control group was decreased (3.52 (3.47–5.52)) when compared to intact (9.90 (9.32–11.32), *p* = 0.0031) and therapeutic (9.76 (9.38–12.90), *p* = 0.0013) groups, which were no different from each other (Figure 3B). At 8 weeks, after SCI the content of slow skeletal fibers in the intact slow soleus muscles was 92% (Figure 3A,D) and decreased in the control group (45%, *p* = 0.0011736) 8 weeks after SCI (Figure 3A’,D). In treated animals, the percentage of slow muscle fibers in *m. soleus* was 63% (Figure 3A’’) and did not differ from that of intact animals (Figure 3D). Morphometric analysis of muscle fibers area (μm^2^) in the soleus muscles revealed that the average area of muscle fibers in the control mini pigs (1058.142 ± 158.093 μm^2^) was lower than in the intact animals (1366.473 ± 139.559 μm^2^) (*p* = 0.1304793). In the therapeutic group, the average area of muscle fibers in the soleus (1293.308 ± 189.384 μm^2^) did not differ from that of the intact group (Figure 3C). Thus, the improvement of motor activity, inhibition of skeletal muscle atrophy, and preservation of soleus muscle phenotype) suggest that autologous leucoconcentrate enriched with *vegf*, *gdnf*, and *ncam* has a positive effect on the recovery of skeletal muscles of hind limbs in mini pigs with SCI.

### 3.2. Lumbar Spinal Cord and Tibial Nerve Plasticity

Immunofluorescence study of the lumbar spinal cord and morphometric analysis of myelinated nerve fibers of both tibial nerves were conducted in relation to the hind limbs motor function recovery in the mini pigs at 8 weeks after SCI.

#### 3.2.1. Motor Neurons

**Figure 4:**
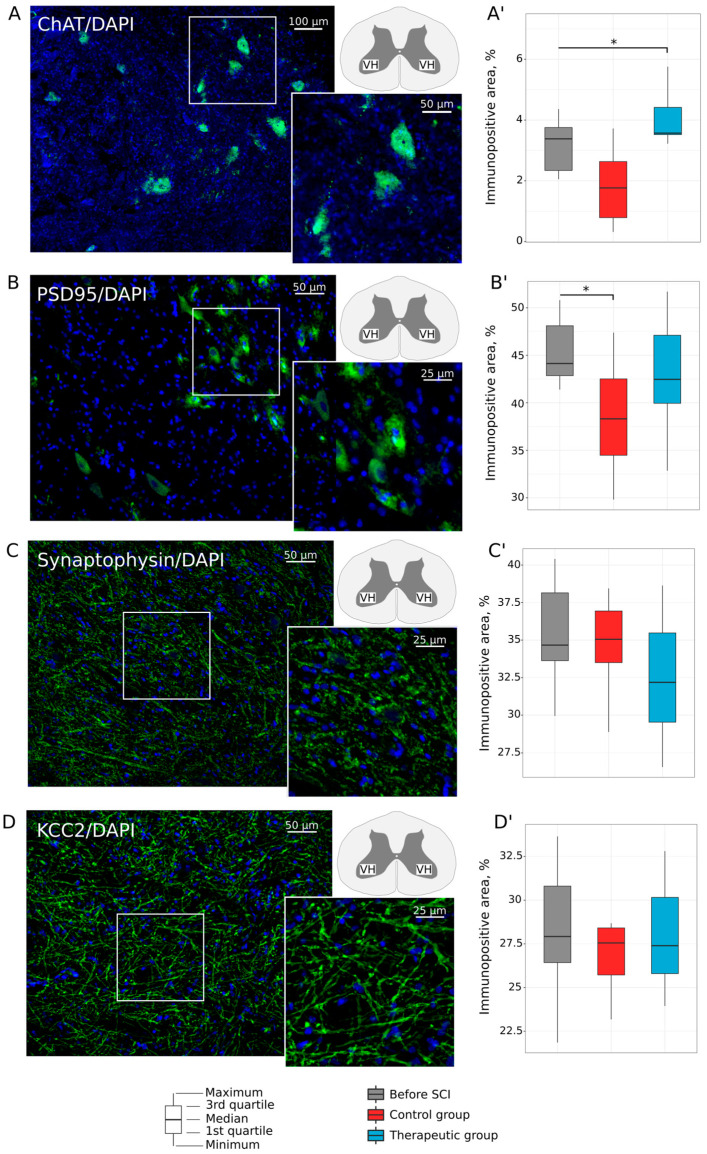
Immunofluorescence staining of the ventral horns of the lumbar spinal cord in mini pigs 8 weeks after low thoracic spinal cord injury (SCI). (**A**) Immunopositive reaction with an antibody against a choline acetyltransferase (Chat) (green glow) in an intact animal. (**A′**) Morphometric analysis demonstrates relative Chat-positive area in the experimental groups. (**B**) Immunopositive reaction with an antibody against a postsynaptic density protein of 95 kDa (PSD95) (green glow) in an intact animal. (**B′**) Morphometric analysis demonstrates relative PSD95-positive area in the experimental groups. (**C**) Immunopositive reaction with an antibody against a synaptic vesicle protein synaptophysin (green glow) in an intact animal. (**C′**) Morphometric analysis demonstrates relative synaptophysin-positive area in the experimental groups. (**D**) Immunopositive reaction with an antibody against a potassium-chloride cotransporter protein (KCC2) (green glow) in an intact animal. (**D′**) Morphometric analysis demonstrates relative KCC2-positive area in the experimental groups. Nuclei were counterstained with DAPI (blue glow). Inserts demonstrate the zooming part of images. The squares inserted in the schematic transverse spinal cord indicate the areas used for immunofluorescence analysis, VH—ventral horn. Data are visualized using box plots, *—*p* < 0.05.

Expression of Chat, PSD95, Synaptophysin, and KCC2 was evaluated in the lumbar motor neurons. Analysis of Chat immunoreactivity in the ventral horns of the lumbar spinal cord revealed that the relative immunopositive area for Chat was higher in the treated animals (3.569 (3.520; 4.416)%, *p* = 0.0173) in comparison with control mini pigs (1.766 (0.789; 2.632)%) and was similar to that of the intact (3.382 (2.343; 3.752)%) group (Figure 4A). The decreased immunoreactivity for PSD95 (Figure 4B) as well was found in the control animals (38.308 (34.474; 42.513)%) when compared to intact mini pigs (44.132 (42.856; 48.104)%) (*p* = 0.0235). In the therapeutic group, expression of PSD95 (42.463 (39.947; 47.107)%) was not different from that in the intact group. Analysis of the relative immunopositive area for synaptophysin (Figure 4C) and KCC2 (Figure 4D) in the ventral horns of the lumbar spinal cord did not reveal essential changes in the therapeutic and control groups when compared to the intact group.

#### 3.2.2. Neuroglial Cells

**Figure 5:**
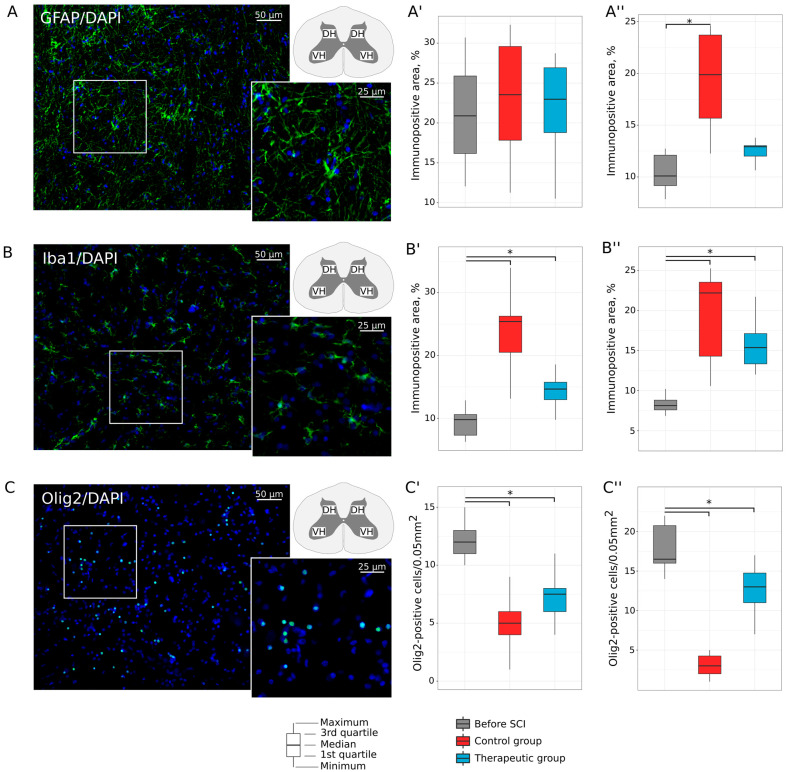
Immunofluorescence staining of the ventral and dorsal horns of the lumbar spinal cord in mini pigs 8 weeks after low thoracic spinal cord injury (SCI). (**A**) Immunopositive reaction with an antibody against a glial fibrillary acidic protein (GFAP) (green glow) in an intact animal. (**A′**,**A″**) Morphometric analysis demonstrates relative GFAP-positive area in the ventral and dorsal horns, correspondingly, in the experimental groups. (**B**) Immunopositive reaction with an antibody against an ionized calcium binding adaptor molecule 1 (Iba1) (green glow) in an intact animal. (**B′**,**B″**) Morphometric analysis demonstrates relative Iba1-positive area in the ventral and dorsal horns, correspondingly, in the experimental groups. (**C**) Immunopositive reaction with an antibody against an oligodendrocyte transcription factor (Olig2) (green glow) in an intact animal. (**C′**,**C″**) Morphometric analysis demonstrates the number of Olig2-positive nuclei in the ventral and dorsal horns, correspondingly, in the experimental groups. Nuclei were counterstained with DAPI (blue glow). Inserts demonstrate the zooming part of images. The squares inserted in the schematic transverse spinal cord indicate the areas used for immunofluorescence analysis, VH—ventral horn and DH—dorsal horn. Data are visualized using box plots, *—*p* < 0.05.

Remodeling of astrocytes, oligodendroglial, and microglial cells was investigated in the ventral and dorsal horns in the lumbar spinal cord of the experimental animals. Analysis of GFAP expression found an increase in relative GFAP-positive area in dorsal horns in control animals (19.871 (15.677; 23.700)%, *p* = 0.002), in comparison with intact animals (10.095 (9.165; 12.101)). In the therapeutic group, GFAP-positive area was not different in the ventral and dorsal horns relative to the intact group (Figure 5A). Evaluation of Iba1 expression demonstrated increased Iba1-positive area in control and treated mini pigs both in ventral (25.409 (20.503; 26.254)%, *p* = 0.0001; 14.657 (12.976; 15.744)%, *p* = 0.003) and dorsal (22.190(14.292; 23.533)%, *p* = 0.001; 15.386 (13.332; 17.112)%, *p* = 0.0006) horns when compared to intact animals (9.802 (7.347; 10.611)% and 8.118 (7.580; 8.794)%) (Figure 5B). The number of Olig2-positive nuclei in intact mini pigs was 12.0 (11.0; 13.0) in the ventral and 16.5 (16.0; 20.75) in the dorsal horns. In the ventral horns, the number of oligodendroglial cells was decreased in the control (5.0 (4.0; 6.0), *p* = 0.0001) and treated (7.5 (6.0; 8.0), *p* = 0.0055) mini pigs. In the dorsal horns, the decreased number of the Olig2-positive nuclei was shown in the control group 3.0 (2.0; 4.25) when compared both with intact (*p* = 0.0001) and therapeutic group 13.0 (11.0; 14.75) (*p* = 0.0006) (Figure 5C).

#### 3.2.3. Tibial Nerve Myelinated Fibers

**Figure 6:**
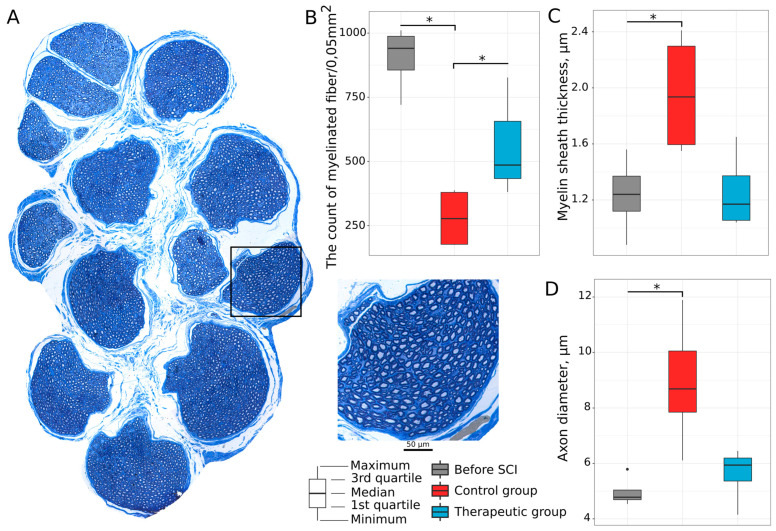
Preservation of the tibial nerve myelinated fibers in mini pigs 8 weeks after low thoracic spinal cord injury (SCI). (**A**) Semi-thin cross-section of the tibial nerve stained with methylene blue dye in an intact animal. (**B**) Number of the myelinated fibers. (**C**) Thickness of myelin sheath (μm) in the myelinated fibers. (**D**) Axon diameter (μm) of the myelinated fibers. Data are visualized using box plots, *—*p* < 0.05.

Myelinated fibers in both tibial nerves (Figure 6A) were studied 8 weeks after SCI with respect to axons of the lumbar spinal cord motor neurons innervating soleus muscles. The count of myelinated fibers found a decrease in the nerve fibers in the control group (277.180 (177.037; 379.340)) when compared to the intact (940.625 (856.108; 987.652), *p* = 0.0013749) and therapeutic (485.850 (433.360; 656.345), *p* = 0.0551393) groups (Figure 6B). No difference was observed between intact and treated animals. The thickness of myelin sheath (1.935 (1.595; 2.298)) and axon diameter (8.69 (7.850; 10.050)) in the control mini pigs was increased when compared to the intact mini pigs (1.240 (1.120; 1.370), *p* = 0.0209560 and 4.78 (4.690; 5.040), *p* = 0.0033348), correspondingly (Figure 6C,D). In the therapeutic group, the thickness of the myelin sheath was 1.170 (1.055; 1.372), and the axon diameter was 5.94 (5.365; 6.195), which was not differ from the intact group (Figure 6C,D). Thus, the preserved number of myelinated fibers and their morphology (axon diameter and thickness of myelin sheath) in the treated animals suggest that autologous leucoconcentrate producing recombinant VEGF, GDNF, and NCAM may affect lumbar motor neurons, dorsal root ganglion neurons, and their neurites in the tibial nerve.

Thus, the study of lumbar spinal cord, peripheral nerves, and hind limb skeletal muscles in mini pigs with low thoracic SCI revealed the essential changes in the structures distant to the primary lesion site, which may be considered as important therapeutic targets in the SCI treatment. The complex analysis of the obtained data demonstrated correlations between the positive changes in the lumbar spinal cord (positive remodeling of the neuroglia and increased expression of synaptic proteins), tibial nerves (preservation of the number and morphological characteristics of the myelinated nerve fibers) and hind limbs skeletal muscles (prevention of soleus muscles atrophy and sparing its phenotype) in the condition of the treatment with GEL.

## 4. Discussion

Spinal cord injury results in the loss of motor, sensory, and autonomic functions of various organs and has a lifelong devastating impact on independence and activities of daily living. Pathological processes following SCI include mass death of spinal cord cells, nerve fibers tears, hemorrhages, and ischaemic and inflammatory lesions, which subsequently form cavities and cysts. Neuroinflammation (activation of astroglia and microglia and infiltration of myeloid cells) is a key factor of the secondary injury involving the entire spinal cord in the pathologic process [1,41]. The post-traumatic spinal cord remodeling takes place not only at the epicenter of the injury but also in large areas around the epicenter [10,42]. Spatiotemporal neurodegeneration in rostral and caudal regions of the spinal cord distant from the primary injury suggests that these areas should be considered as important therapeutic targets, particularly when analyzing the effectiveness of SCI therapies [14,43]. However, the effect of the therapies for SCI has generally been considered mainly regarding the therapeutic targets at the epicenter of the lesion and in the adjacent segments [37]. 

Earlier, we studied the effect of autologous genetically enriched leucoconcentrate producing recombinant VEGF, GDNF, and NCAM on molecular and cellular changes in the epicenter of the injury [33]. Cavitation in the grey and white matter, astrogliosis, decreased number of oligodendroglial cells, and affected expression of the synaptic proteins were revealed at 60 days after SCI at the Th8-Th9 level in SCI mini pigs. Intravenous infusion of the autologous genetically enriched leucoconcentrate had a positive effect on the sparing of the grey matter, recovery of synaptic protein expression, prevention of astrogliosis, and growth of regenerating axons accompanied by a higher number of oligodendrocytes in the rostral and caudal segments near the epicenter of the injury. 

The positive effect of GEL-VGN auto-infusion four hours after SCI modeling was based on the neuroprotective action of VEGF [44], GDNF [45,46], and NCAM [37,47,48,49] on affected nervous tissue. The neuroprotective action of VEGF promotes the survival of neuronal cells mediated via the activation of high-affinity receptor tyrosine kinases by the activation of mitogen-activated protein kinase (MAPK) [50,51] and phosphatidylinositol 3-kinase (PI3K) signaling cascades [52,53]. One of the key functions of GDNF is the maintenance of neuron–neuron and neuron–target tissue interactions and prevention of cell death [54]. GDNF triggers phosphorylation of the RET (rearranged during transfection) receptor tyrosine kinase responsible for activation of the MAPK and/or PI3K pathway [55,56]. NCAM (CD56) is expressed on the surface of neurons and neuroglia cells. NCAM-mediated intercellular interactions in neuroontogenesis and post-traumatic regeneration ensure neuronal survival, directed neurite growth, and synaptogenesis [49]. NCAM recruits non-receptor Fyn tyrosine kinase, leading to MAPK and transcription factor CREB (cAMP response element binding protein) activation [47]. NCAM also serves as a signaling co-receptor for GDNF [57].

Based on in vitro studies, we confirmed effective expression of *vegf165, gdnf*, and *ncam1* in genetically modified leucocytes 72 h after transduction with adenoviral vectors [33,37]. Delivery of *vegf165, gdnf*, and *ncam1* with autologous leucocytes proposes effective production of the recombinant molecules in about 2–3 weeks, which is limited by the expression of adenovirus vectors [38,58]. After intravenous infusion of GEL-VGN, recombinant VEGF, GDNF, and NCAM produced by gene-modified leucocytes in the bloodstream may reach the injury site via the impaired blood–brain barrier after SCI [59]. The role of NCAM in the GEL-VGN is also proposed to stimulate migration of the gene-modified leucocytes into the damaged spinal cord and provide the local production of the neurotrophic factors [60]. Active expression of the transgenes in the acute phase of SCI may reduce the negative consequences of the neurotrauma and stimulate post-traumatic spinal cord regeneration. However, the role of each therapeutic molecule in this combination has to be further explored.

In this work, we expanded our previous study and investigated dysfunction of the lumbar spinal cord in mini pigs with low thoracic SCI. We specifically evaluated the effect of the autologous genetically enriched leucoconcentrate on the morphological and functional restoration of the lumbar spinal cord in regard to the condition of the myelinated fibers of the peripheral nerves (*n. tibialis*) and the skeletal muscles of the hind limbs (*m. soleus*) innervated by tibial nerve branches.

The rationality of the study is based on our hypothesis that intravenous infusion of the autologous genetically enriched leucoconcentrate may affect not only nervous tissue at the epicenter of the lesion by paracrine action of the recombinant VEGF, GDNF, and NCAM produced by genetically modified leucocytes migrated into the damaged region, but also the areas distant from the injury site via the endocrine mechanism of action of the recombinant therapeutic molecules secreted by the genetically modified leucocytes into the blood stream.

### 4.1. Lumbar Spinal Cord

The negative consequences reached the lumbar spinal cord about 60 days after low thoracic SCI in control animals in the control group with no therapy. Notably, the pathological distribution of neuroglial cells found in the lumbar spinal cord was similar to that near the epicenter of the injury [33]. Immunofluorescence analysis of the GFAP expression revealed an increase in the immunopositive area occupied by astrocytes in the dorsal horns of the lumbar spinal cord. The Iba1-immunopositive area corresponding to the localization of microglial cells was increased in both the ventral and dorsal horns. The number of oligodendroglial Olig2-positive cells significantly decreased in the ventral and dorsal horns of the lumbar spinal cord. In treated mini pigs, infusion of the autologous genetically enriched leucoconcentrate producing recombinant VEGF, GDNF, and NCAM positively affected the remodeling of microglial cells in the ventral horns and astrocytes and oligodendroglial cells in the dorsal horns of the lumbar spinal cord. Immunofluorescence analysis of synaptic protein expression in the ventral horns near the neurotrauma revealed a reduced immunopositive area for synaptophysin and PSD95. At the same time, in the lumbar part, we found restoration of synaptophysin expression and still-reduced PSD95 expression. The autologous genetically enriched leucoconcentrate increased the immunopositive area for PSD95 and Chat in the lumbar spinal cord. Thus, in the lumbar spinal cord of SCI mini pigs at the low thoracic level, molecular and cellular changes demonstrated the efficacy of the autologous genetically enriched leucoconcentrate producing recombinant VEGF, GDNF, and NCAM to overcome these negative consequences (recovery of synaptic protein expression by motor neurons and positive neuroglial cell remodeling).

### 4.2. Peripheral Nerves

After SCI, peripheral nerves carrying motor, sensory, and autonomic axons have severe morphological disorders [61,62]. In our study, morphometric analysis of the tibial fascicle of the sciatic nerve in control mini pigs revealed a decrease in the number of myelinated fibers and an increase in myelin thickness and nerve fiber axon diameter. Such changes may be associated with degeneration of the sensory and sympathetic fibers due to direct damage of the dorsal and lateral columns of the spinal cord at the low thoracic level. The possible preservation of the motor axons in control animals was evident by sparing motor neurons in the lumbar region 60 days after SCI, which had an equal expression of KCC2 compared to intact mini pigs. In the therapeutic group, intravenous infusion of the autologous genetically enriched leucoconcentrate producing recombinant VEGF, GDNF, and NCAM positively affected the morphological sparing of tibial nerves myelinated fibers.

### 4.3. Hind Limb Skeletal Muscle

Disrupted connections between central neurons of the pyramidal and extrapyramidal systems and motor neurons of the spinal cord directly reflect the condition of the hind limbs skeletal muscles innervated by motor neurons. After low thoracic SCI in control mini pigs without therapy, the PTIBS score was equal to 1 point, and by 60 days, it had reached 2.0 points. Behavioral results were consistent with electrophysiological (polyphasic M-response associated with increased duration and amplitude) and morphological (decreased muscle weight, decreased average skeletal muscle fiber area, and a number of slow skeletal muscle fibers) characteristics of the soleus muscle. In mini pigs treated with autologous genetically enriched leucoconcentrate, better recovery of voluntary locomotion scored 3.0 by the PTIBS test was accompanied by prevention of soleus muscle atrophy and slow to fast transformation of muscle fibers, which suggests the effectiveness of the neurotrophic control of skeletal muscle fibers via axons of the lumbar motor neurons. The observed recovery of the hind limbs motor function was strongly supported by the beneficial effect of autologous genetically enriched leucoconcentrate on: (1) the damaged spinal cord at the thoracic level (sparing of the grey matter, inhibition of astrogliosis, growth of axons accompanied by increased numbers of oligodendroglial cells), as was shown earlier [33]; (2) the lumbar spinal cord (recovery of PSD95 and Chat expression in motor neurons, positive neuroglial cell remodeling); and (3) the tibial nerves morphological preservation. It is evident that regeneration of axons through the epicenter of injury, restoration of motor neurons of the lumbar region and preservation of their axons providing neurotrophic control of the skeletal muscles generally results in improved locomotion of the hind limbs, which is in line with inhibition of skeletal muscle atrophy (preservation of raw muscle weight and muscle fiber area) and sparing the muscle phenotype (slow and fast muscle fiber content).

The recombinant VEGF, GDNF, and NCAM secreted into the bloodstream may have a positive effect on the neuromuscular junctions as well. Thus, it was shown that genetically modified human mesenchymal stem cells, simultaneously overexpressing GDNF and VEGF, when injected intramuscularly into rats with a model of amyotrophic lateral sclerosis (ALS), maintain the neuromuscular synapse structure and prolong animal life [63]. In another study, ALS mice injected intramuscularly with a combination of two adenoviral vectors carrying cDNA of VEGF and angiogenin demonstrated a delay in the manifestation of the disease, higher motor activity, and increased lifespan [64]. Intravenous injection of human umbilical cord blood mononuclear cells simultaneously co-transduced with Ad5 carrying cDNA of *vegf165, gdnf*, and *ncam1* resulted in a prominent increase in life span and improved performance in behavioral tests in ALS mice [60]. In the model of hypogravity on Earth, administration of a composition of three adenoviral vectors carrying *vegf165, gdnf*, and *ncam1* into mice hind limbs skeletal muscles before the hind limbs unloading had a positive effect on myelinated fibers in the anterior spinocerebellar tract and sciatic nerve [65]. In our study, the recombinant VEGF, GDNF, and NCAM via the endocrine mechanism may have affected post-traumatic spinal cord recovery at lumbar spine and neuromuscular junction levels as well.

Employment of autologous leucocytes in gene therapy for the temporary production of biologically active molecules by demand represents one of the breakthrough directions in gene therapy for SCI [37]. Recombinant therapeutic molecules produced by genetically modified leucocytes at the site of injury may have a local effect (paracrine) on neuron survival, regrowth of the axons, and recovery of neural circuitry, with a simultaneously positive systemic (endocrine) effect on the preservation of lumbar motor neurons, their axons, and neuromuscular junctions. Standing with the efficacy of the SCI treatment obtained in this study, we provided evidence that auto-infusion of genetically enriched leucoconcentrate producing recombinant neurotrophic factors and neuronal cell adhesion molecules is a novel, potentially successful approach for multitarget treatment of SCI. Meanwhile, the exploratory results obtained in the study indicate the importance of performing further experiments with a higher number and different gender of large animals with anatomical, physiological, and biochemical characteristics close to humans in preclinical investigations. The complex nature of the GEL-VGN raises important questions about dose, mode of delivery, pharmacokinetics of the therapeutic molecules, etc. Further research with human blood is needed to develop a protocol for the preparation of genetically enriched leucoconcentrate before its translation to clinical trials.

## 5. Conclusions

This research aimed to test the hypothesis that intravenous infusion of an autologous leucoconcentrate enriched with therapeutic genes is a potentially successful approach for multitarget treatment of thoracic SCI in clinical practice. Our results demonstrate that in mini pigs with low thoracic SCI, autologous genetically enriched leucoconcentrate producing recombinant neurotrophic factors (VEGF and GDNF) and neural cell adhesion molecules (NCAM) affect not only the epicenter of injury but also remote structures, including lumbar spinal cord, peripheral nerves, and hind limbs skeletal muscles. We believe that the simple, safe, and economical method of the genetically enriched leucoconcentrate preparation from the patient’s peripheral blood and chimeric adenoviral vectors (Ad5/35F) carrying cDNA of the therapeutic genes might represent a novel avenue for future personalized precision gene therapy research for other CNS disorders, including neurodegenerative diseases and stroke.

## Figures and Tables

**Table 1:** Antibodies used in immunofluorescence staining.

Antibody against:	Host	Dilution
Choline Acetyltransferase (ChAT)	Rabbit	1:100
Glial fibrillary acidic protein (GFAP)	Mouse	1:200
Ionized calcium binding adaptor molecule 1 (Iba1)	Rabbit	1:150
The K+–Cl− cotransporter isoform 2 (KCC2)	Rabbit	1:100
Oligodendrocyte transcription factor 2 (Olig2)	Rabbit	1:100
Postsynaptic density protein 95 kDa (PSD95)	Rabbit	1:200
Slow Skeletal Myosin Heavy chain	Rabbit	1:100
Synaptophysin	Rabbit	1:100
Mouse IgG conjugated with Alexa 488	Donkey	1:200
Rabbit IgG conjugated with Alexa 488	Donkey	1:200

## Data Availability

The data presented in this study are available on request from the corresponding author.
